# Sepsis-induced Cardiomyopathy

**DOI:** 10.2174/157340311798220494

**Published:** 2011-08

**Authors:** Francisco J Romero-Bermejo, Manuel Ruiz-Bailen, Julián Gil-Cebrian, María J Huertos-Ranchal

**Affiliations:** 1 Intensive Care Unit, Critical Care and Emergency Department. Puerto Real University Hospital; 2 Intensive Care Unit, Critical Care and Emergency Department, Medical-Surgical University Hospital of the Jaen Hospital Complex

**Keywords:** Myocardial dysfunction, cardiac depression, heart failure, sepsis, severe sepsis, septic shock.

## Abstract

Myocardial dysfunction is one of the main predictors of poor outcome in septic patients, with mortality rates next to 70%. During the sepsis-induced myocardial dysfunction, both ventricles can dilate and diminish its ejection fraction, having less response to fluid resuscitation and catecholamines, but typically is assumed to be reversible within 7-10 days. In the last 30 years, It´s being subject of substantial research; however no explanation of its etiopathogenesis or effective treatment have been proved yet. The aim of this manuscript is to review on the most relevant aspects of the sepsis-induced myocardial dysfunction, discuss its clinical presentation, pathophysiology, etiopathogenesis, diagnostic tools and therapeutic strategies proposed in recent years.

## INTRODUCTION

Severe sepsis and septic shock are the main cause of death in non-cardiac Intensive Care Units (ICU), with unacceptably high mortality rates [1, 2]. In 1995, the Angus group estimated a death rate of 38.6% in the United States in a cohort of 751.000 cases of severe sepsis [3]. During the years 1993-2003 Dombrovskiy group aimed to a rapid increase in hospitalizations for severe sepsis and iits mortality, with an annual growth of 8.2% (p <0.001) and 5.6% (p <0.001) respectively, and crude mortality rate approaching 50% with significant differences with increasing patient age [4]. 

The study "*Sepsis Occurrence in Acutelly ill Patients*” (SOAP), involved 21 European countries and enrolled 3.147 septic patients for 15 days in May 2002. Mortality rates were 27% in septic patients and more than 50% of patients with septic shock [5]. Other European studies found similar mortality rates [6-9]. Encouraging data recently obtained from the authors of the Surviving Sepsis Campaign, after two years since the introduction of their protocols, showed to reduce hospital mortality from sepsis by 37% to 30.8% (p = 0.001) [10]. However, this mortality rate remains very high. 

Sepsis-induced myocardial dysfunction (SIMD) is one of the major predictors of morbidity and mortality of sepsis. It is present in more than 40% of cases of sepsis [11] and its appearance can increase the mortality rate up to 70% [12]. Nearly 30 years of research on SIMD have not been sufficient to improve the results substantially and, moreover, there are controversies about its pathophysiology and treatment strategies, many still in the experimental period, but the evidence suggests that seems to be different from some of the recommendations of the worldwide distributed guide Surviving Sepsis Campaign, provided a major challenge. 

The aim of this paper is to review about the most important aspects of the SIMD.

## BACKGROUND

Reversible myocardial dysfunction (RMD) was first described in a canine experimental model by Heyndrickx and coworkers at 1.975 [13]. The phenomenon of RMD generated after the induction of transient ischemia in coronary arteries without producing necrosis was called "*stunned myocardium*" in 1.982. It was self-limited but could lead to decreased ventricular compliance [14]. 

Clowes and coworkers in 1966, with a cohort of patients with diffuse visceral fulminating peritonitis [15], suggested that cardiovascular involvement associated with sepsis was based on two patterns in the clinical examination. Initially, patients had a hyperdynamic phase (“*warm shock*"), which still had extremities hot but with a bounding pulse (increased cardiac output and decreased systemic vascular resistance). Later, the hypodinamyc stage ocurred ("*cold shock*"), in which the patient had a thready pulse, signs of peripheral hypoperfusion, organ failure and ultimately died (systemic vascular resistance increased to compensate for reduced cardiac output) [16]. 

With the introduction of hemodynamic monitoring with pulmonary artery catheter, the existence of myocardial depression in patients with septic shock was confirmed, showing depressed cardiac response to volume infusion [17, 18]. The extrapolation of the results of these studies was limited by having no control and can not rule out other possible causes of depressed cardiac response to volume infusion. With the addition of thermodilution study, it was demonstrated that patients who underwent correct fluid resuscitation had only a hyperdynamic state (with hot extremities, high cardiac output and low systemic vascular resistance). The hyodinamyc stage was considered to be due to an inadequate fluid resuscitation [19, 20]. 

The concept of SIMD emerged from the study of Parker and coworkers at 1.984 [21]. They demonstrated, using radionuclide ventriculography and simultaneous study of cardiac output (CO) by thermodilution, the existence of myocardial depression in a cohort of 20 patients with septic shock. Patients who survived (13 of 20), had an initial left ventricle ejection fraction (LVEF) less than 40% (p <0.005), with mean end-systolic volume and end-diastolic volume increased substantially with a normal stroke volume (SV). These changes were sustained for as long as four days and then gradually returned to normality by 10 days after the onset of shock. Paradoxically, nonsurvivors had normal LVEF and ventricular volumes. Other studies supported the development of RMD in noncardiac pathology [22, 23], having shown that this phenomenon occurs in a wide range of critically illness (Table **1**)[24]. 

## PATHOPHYSIOLOGY

**Fig. (1):**
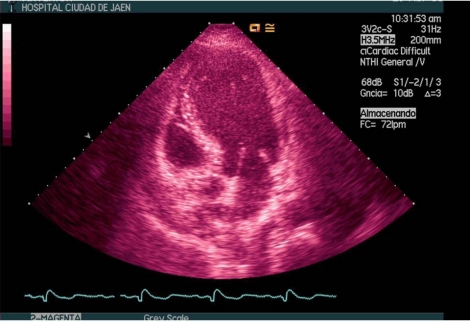
Echocardiographic image of a patient in septic shock secondary to fecaloid peritonitis. The left ventricle shows a pathological remodeling similar to that generated in an anterior wall acute myocardial infarction.

**Fig. (2):**
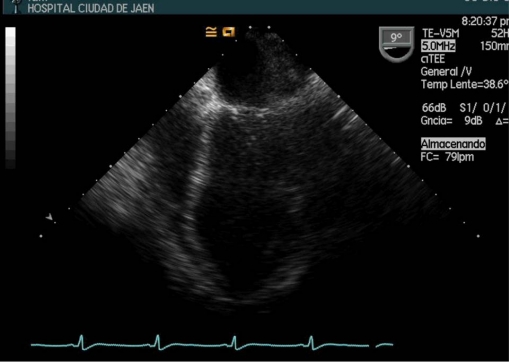
Transesophageal image projection which displays the left ventricle of a 18-year-old female patient with urinary sepsis by *E. colli*. The arrow points to "*ballooning*" septoapical segment.

In 10-20% of patients with refractory hypotension (need for dopamine >15 mg / kg per min or norepinephrine >0.25 mg / kg per min to maintain mean blood pressure above 60 mmHg or 80 mmHg if previous hypertension), exists high probability that CO was diminished by the presence of severe myocardial dysfunction [1, 3, 25]. During the SIMD both ventricles can dilate and diminish its ejection fraction, having less response to fluid resuscitation and to increase CO despite starting treatment with catecholamines. The degree of SIMD is very variable, ranging from mild to severe [26, 27]. Sometimes the degree of myocardial depression may be so severe that imitates a cardiogenic shock [28], but typically is assumed to be reversible within 7-10 days [21, 29, 30] (Figures **1-2**). 

### Left Ventricle Impairment

After the study of Parker and coworkers [21], left ventricular (LV) dilatation was considered to have some protective effects in septic patients suggesting that it compensated the decreased CO and ventricular contractility by the Frank-Starling mechanism and also, and for its reversibility in 7-10 days, it was associated with better prognosis. Subsequent studies have not confirmed these compensatory mechanisms [8, 9, 30-32]. In 2004 Charpentier group performed an echocardiographic study with a cohort of 34 patients with severe sepsis or septic shock, of whom 15 had SIMD (44%) [8]. These patients with SIMD (defined as fractional area contraction <50%), received more fluid charges and had higher mortality (47% versus 16% in control group, p = 0.04). However, they also had higher scores on the scale Simplified Acute Physiologic Score II, a greater number of organ failure and need for mechanical ventilation, but these data were not statistically significant. Interestingly, no patient developed ventricular dilation. In a study of Vieillard-Baron and coworkers [9], with a cohort of 67 patients with septic shock connected to mechanical ventilation who survived >48 hours, transient LV dysfunction was not associated with worse prognosis. Bouhemad and coworkers [33], did a follow-up echocardiography study with 45 patients with septic shock focusing on acute changes in LV dimensions. In this study, LV dilatation in the acute phase of septic shock reached 30%, lower than dilatation rate found in the study of Parker (100% of patients had dilated LV) [21], possibly due to not have used aggressive fluid therapy in these patients and initiate vasoactive therapy early. They also found that the LV dilatation was presented in patients with LV dysfunction and that diastolic dysfunction impaired relaxation type didn´t change its dimensions. 

Recently, Zanotti-Cavazzoni group studied the impact of SIMD on the LV diameter, hemodynamic parameters and survival in a murine experimental model [34]. In septic mice (n = 24), 37% had dilated LV (end-diastolic diameter increase in ≥ 5% from baseline values). With resuscitation SV and CO improved to a greater extent in dilators than non-dilators (both p < 0.05). Survival at 72 hours improved significantly in dilators (88% vs. 40%, p = 0.01). 

### Right Ventricle Impairment

Phenomena similar to those mentioned in LV have been described in the right ventricle (RV) [35]. In contrast to LV function, the RV usually has a high afterload in sepsis by increased pulmonary vascular resistance due to Acute Lung Injury or Adult Respiratory Distress Syndrome, which can lead to decreased RV CO [35]. In addition, the RV does not usually compensate for acute elevations in afterload, developing cardiogenic shock in situations as massive pulmonary embolism [37]. Vincent and coworkers [38] studied 68 patients with septic shock and found that survivors group had higher arterial pressure and right atrial pressure, higher SV and higher RV ejection fraction (RVEF) than had the patients who died; therefore the decrease in RVEF was associated with poor prognosis. Vieillard-Baron and coworkers [9], only observed RV dilatation in 24% of patients and there were not statistically significant differences in RVEF of patients without RV dilation (Table **2**). 

## DIASTOLIC FUNCTION

The LV diastolic function (compliance) can be studied using transmitral and pulmonary veins Doppler-pulsed and Doppler tissue imaging echocardiography. The transesophageal study has proven to be better than the transthoracic one due to a higher quality of images and the possibility of complete analysis of pulmonary veins flow (Image** 3**)[36]. Few studies have been conducted of sepsis-induced diastolic dysfunction and all these have small sample size [40-42], or are experimental with animal [34]. SIMD is a continuum from isolated diastolic dysfunction to both diastolic and systolic ventricular failure [40]. In the study of Munt and coworkers [41], diastolic function was studied 24 patients with severe sepsis. Nonsurvivors had more pronounced diastolic dysfunction than survivors, showing a pattern of abnormal ventricular relaxation with a peak filling rate normalized to mitral SV (E/VTI) 4.7 vs. 8.8 (p = 0.04), deceleration time 235 ms vs. 182 ms (p = 0.02) and E/A with no significant differences. In multivariate analysis, in addition to the APACHE II score, only the deceleration time was an independent predictor of mortality (p <0.004). Jafri and coworkers on a case-control study [42] with 23 patients diagnosed with severe sepsis and septic shock, found that septic patients had diastolic dysfunction with increased reliance on atrial systolic contribution to diastolic filling. In patients with diastolic dysfunction prior to the SIMD, difficult weaning from mechanical ventilation and increased need for inotropic support could be predicted [43, 44]. Mathru and coworkers [45], in a case-control pilot study with a group of 11 young people (aged 30 ± 6 years), previously healthy and endotoxin-challenged volunteers who had not received empirical volume loading, despite stating that knowledge of diastolic function status could be very helpful to predict how a septic patient would respond to changes in preload, didn´t find statistically significant differences in diastolic function with the control group. The authors found significant increase in wave A and E/A ratio after-endotoxin infusion and no significant changes from baseline in the E wave (p <0.05). Within the limitations of this study, the most important is that in a human sepsis model without confounding variables encountered in clinical sepsis (comorbilities, advanced mean age, genetic polymorphisms, organ dysfunction, polymicrobial infections, etc.), the extrapolation of the results would be difficult. 

## ETIOPATHOGENESIS

Many pathological findings were found in the SIMD, including genetically [46], all possible mechanisms both those that affect the environment of cardiomyocytes, as the myocardial cell itself have been investigated. None of the findings is conclusive in itself, so it is likely that these alterations of contractility and myocardial function in sepsis were a result of the interaction of many factors that normally regulate the contractile apparatus and its system of signals. 

### Extracellular Mechanisms

#### Alterations of Myocardial Flow - Myocardial Ischemia

Early theories of myocardial dysfunction in sepsis were based on global myocardial ischemia. Several canine experimental studies with endotoxemic shock so hypothesized it [47,48]. This theory was refuted after Cunnion and coworkers study, with a group of seven patients with septic shock who underwent thermodilution coronary sinus catheterization and demostrated that the coronary flow was similar or even higher in patients with SIMD [49]. Other studies supported these results to find a marked coronary vasodilation in septic patients with no elevation of myocardial lactate production [50]. In sepsis models using spectroscopyc magnetic resonance, no deterioration in the metabolism of high energy phosphates has been shown, which supports the hypothesis of low involvement in energy metabolism of the septic patient myocyte [51]. 

#### Alterations in the Microcirculation

The microcirculation undergoes major changes during sepsis. It was thought that could be changes in the distribution of flow that conditioned localized areas of ischemia, and even that it was the reason for the appearance of elevated troponin levels that have been found occasionally, in correlation with the severity of cardiac dysfunction [52]. Nowadays it is thought that plasmatic troponin elevation is likely due to an increase in membrane permeability induced by myocardial cytokines, although it is a matter still under discussion. Alterations in the distribution of coronary flow [53], could be caused by endothelial swelling and intravascular fibrin deposits that fail to occlude the light [51]. Migration and activation of circulating neutrophils into the interstitium also were found [54]. However, no cellular hypoxia has been confirmed in animal models [55]. All this makes these mechanisms appear to be low important in the onset of SIMD. 

#### Myocardial Depressant Substances 

Lefer [56] in 1970 and Wangensteen in 1971 [57] suggested and Parrillo, confirmed in 1985 [58] the existence of some depressant substance in an experimental study. Rat cardiomyocytes were infiltrated with serum from patients in septic shock and it showed that there was a decrease of the amplitude and velocity of shortening of these cells in the acute phase of the disease. It was found that many substances that act as mediators, which are elevated in sepsis and septic shock, such as tumor necrosis factor (TNF-α) [59-61], interleukin (IL-1β), and a factor called complement anaphylatoxin (C5a), also have cardiodepressant properties *in vitro* [62, 63]. *In vivo*, myocardial dysfunction in children with meningococcal septic shock has been shown, apparently caused by IL-6 [64]. Interestingly cardiomyocytes can generate in large burns and sepsis a multitude of substances such as TNF-α, IL-1β, IL-6, a substance that induces chemotaxis of neutrophils (CINC-1), macrophages migration inhibitory factor (MIF) [65-67], and High Mobility Group Box (HMGB-1), many of which we cite as probable sources of SIMD. Currently, we still don´t know the meaning of this paradoxical behavior, which myocardiocytes products inhibit its own function. It has been shown in experimental sepsis [58], that a C5a blocking factor substance could reverse the reduction of LV pressures that appeared in the septic patient, and vice versa, the addition of recombinant C5a in a vitro preparation of myocardial fibers of patients septic or healthy, produced major impaired contractility in both, which suggests it may play an important role in the SIMD development. However, it has been shown that isolated rabbit papillary muscles taken during the acute phase of sepsis had a marked decrease in contractility, despite the absence of direct contact with septic plasma, so the role of these mediators is not clear [68.69]. It is probably not one of these substances isolated but a constellation of them which influenced the onset of the SIMD, generally by the release, activation or inhibition of other cellular mediators (calcium homeostasis, nitric oxide production, etc.). It is also quite unlikely that bacterial endotoxin itself *per se* causes direct myocardial depression, as only a minority of septic patients have detectable levels of endotoxin [70]. 

#### Metabolic Changes 

It has been found an accumulation of intramyocardial lipids and glycogen in non-survivors in septic patients [71] and in experimental animals [72]. The hearts of septic patients usually have a net extraction of lactate, while the uptake of glucose, ketone bodies and free fatty acids are diminished [73]. Oxygen consumption and metabolism at rest are increased by up to 30% respect to normal values, but less even in uncomplicated sepsis [74]. However, when the shock progresses and organ failure occur, oxygen consumption and metabolism decrease again. As a result, the prolonged sepsis and since most of the oxygen consumption occurs in the mitochondrial oxidative phosphorylation, ATP production would decrease, which could explain, at least in part, the organ dysfunction of sepsis [75]. Cardiac muscle supports much worse an oxygen debt that skeletal muscle, where these findings were studied, which would do it especially vulnerable. 

#### Autonomic Dysregulation

Some authors have found apoptosis of neurons and glial cells in autonomic centers that control the cardiovascular system [76, 77], possibly induced by chemical mediators, which could cause an inadequate autonomic control of the circulatory system in patients with sepsis or septic shock. It has been found high levels of catecholamines which coexist with an inadequate control of heart and vessels, which may even precede the manifestations of shock [78, 79]. However, they are most likely other factors such as adrenergic stimulation by insufficient ventricular filling or fever. Tachycardia has several adverse effects: lower ventricular filling, increased oxygen requirement and even a specific cardiomyopathy induced by excessive catecholamine stimulation. While this, for some time, increases contractility and heart rate but if it is prolonged can lead to myocardial damage by intracellular calcium overload and cell necrosis [80]. The more autonomic dyscontrol, greater seems the risk of death. The heart rate was a prognostic factor in some studies [81]. 

### Cellular Mechanisms 

In recent years, increased attention on the cellular mechanisms that can lead to myocardial depression in sepsis and septic shock has been focused on the role of many mediators that are altered, and often by stimulation or inhibition of other mediators, may influence the appearance of the SIMD. It is still not clear, but seems possible to attribute a major role to cytokines produced by various cells of the organism (endothelial, epithelial, fibroblasts, or cells related to the immune system such as neutrophils, lymphocytes or macrophages), under very various stimuli (trauma, infection, sepsis, ischemia, etc.), act as messengers between cells often very remote and can alter their function. 

Although clear relationships were found between the appearance of these cytokines in the circulating blood of septic patients and the onset of myocardial depression *in vitro* [82, 83], it hasn´t been possible to find, in studies with many samples [41], clinical improvement by treatment with TNF-α antibodies which had been found in early studies [84]. Neither the treatment with an antagonist of IL-1 receptors in a study of nearly 700 septic patients, placebo-controlled, showed differences in mortality at 28 days [85]. 

#### Calcium Transport 

In isolated cardiomyocytes from animals, it has been found that both endotoxin [86, 87] and cytokines [88], alter or suppress the L channels-dependent calcium flow, possibly through changes in autonomic regulation of this channel. This causes a reduced concentration of calcium intracellular and a decrease in the fiber contractility. In addition, endotoxin opens ATP-dependent potassium channels, thus shortening the duration of the action potential and reduces the calcium imput [89]. Ryanodine receptor density appears to decrease in models of sepsis, reducing the calcium output from sarcoplasmic reticulum (SR) by stimulating calcium come from outside [90-91]. In some models, the effects on this receptor could be reversed by selective inhibition of Nitric Oxyde Sinthetase 2 (NOS-2) [92]. The pilot action of a recombinant TNK-α decreased contractility secondary to a decrease in intracellular calcium, probably through degradation of sphingomyelin to sphingosine, which blocks the ryanodine receptor and prevents the release of calcium from the SR [93]. 

In animal models of sepsis with cecal ligation and puncture, a reduction of up to 46% of the rate of ATP-dependent calcium entry in hypodinámyc sepsis has been found. Since both calcium-ATPase and its main regulatory protein, the phospholamban, control the active calcium reuptake into the SR, it seems that this finding means defects in phosphorylation of proteins of the SR in myocardial dysfunction of late sepsis [94, 95]. In an animal model, a decrease in enzymatic activity of the c-reductases of the electron transport chain and morphological changes in the ultrastructure of mitochondria in hearts of rats in late sepsis have been found. This correlated with lower ATP content in cardiomyocytes. Interestingly, this effect could be mitigated or even not show up, when before the cecal ligation and puncture (CLP), the whole animal was heated to 41-42 º for 15 minutes [96]. This finding was attributed to heat-induced expression of so-called "heat-shock-proteins" as a protective factor. The authors demonstrated that the expression of two of these proteins, Hsp 72, which helps the transfer of newly synthesized proteins into mitochondria and Grp 75 that helps the storage and binding of proteins in the mitochondria - was preserved in the heat shock, compared to controls, as they believed tthat he respiratory chain dysfunction was correlated with clinical deterioration in sepsis and also it could be prevented or improved by the heat shock response [97, 98]. 

#### Myofibrillar Dysfunction 

Some experiments in animals have found a decrease in the density of calcium L-channels in endotoxemic animals [99], or a decreased sensitivity of myofilaments to calcium [100]. Although little is known about the mechanism of these changes, both may be involved in depressed contractility and impairment of systolic function in sepsis. On the other hand, the reduced calcium sensitivity of myofilaments appears to be associated with increased fiber length, and therefore an increased compliance, which would explain the expansion of cavities which appears frequently in the septic hearts [101]. More recently disruption zones in the actin-myosin contractile apparatus of human hearts of septic patients have been reported [102], which could be due to the increased activity of matrix metalloproteinase, because this enzyme can degrade both the contractile apparatus as cytoskeleton [103, 104]. 

#### CD14 Receptors 

The occurrence of myocardial dysfunction after endotoxin action seems to depend on the presence of receptors on the cell wall type Toll-like receptor (TLR) type 4 [105] and CD-14 [106]. The TLRs have been identified as primary receptors of innate immunity which distinguishes between different patterns of pathogens and evoke a rapid innate immune response. The TLR-4 must be present in macrophages, and in a lesser extent on neutrophils, to cause cardiomyocytes dysfunction during endotoxemia. Genetically deficient animals were protected against endotoxin-induced changes [107]. Apparently, its role would be the release of cytokines, since immunoadsorption prevents myocardial dysfunction. It seems that this mechanism would be noticeable only during the first phase of sepsis, but not in the recovery period. The CD-14 is a glycosylphosphatidylinositin-anchored receptor of about 55 kD that binds to lipopolysaccharide (LPS) endotoxin with high affinity and is involved in processes mediated by this. It has been found that CD-14 deficient mice are protected from shock induced by LPS, and transgenic mice that have transferred this human receptor were particularly sensitive to LPS [108, 109]. Comparing CD-14 deprived mice with normals in endotoxemia, the first maintained a normal cardiac function while the others had a decreased ventricular shortening, circumferential shortening velocity and the dP / dT max [110]. However, as the CD-14 has no transmembrane structure, the exact mechanism of action is at present unknown. 

#### β-Adrenergic Receptors

Excessive stimulation of these receptors, or for a long time, sometimes have caused myocardial damage by intracellular calcium overload and even induced cell necrosis. In murine models of sepsis, it was found decreased density of β-adrenergic receptors [111], while others point to other factors such as cytokines, as β-adrenoceptor density is normal [112]. On the other hand, in intracellular level, it has been found in experimental endotoxemic animals decreased levels of stimulant G protein [113], and by contrast, increased expression of inhibitory G protein [114]. This same observation has been found in the myocardium of patients who survived septic shock [115]. This apparent diminished effect of catecholamines has been attributed to its autoxidation by reactive oxygen species, specifically by superoxide, resulting in inactivation [116]. In an animal model of septic shock, superoxide dismutase (SOD) administration simulated the administration of catecholamines and β-adrenergic response. In animal models and human studies, the decrease in adrenergic response was associated with elevated levels of NO [117, 118]. The sympathetic overstimulation in critically ill patients ("*cathecolaminergic storm*"), mainly affects the heart and can lead to impaired diastolic function, tachycardia and tachyarrhythmias, myocardial ischemia, stunning, apoptosis and necrosis [119]. 

#### MAPK Signaling Cascades 

A large part of the extracellular stimuli recognized by mammalian cells involves a complex signaling molecules in the center of which are the so-called mitogen-activated protein kinases (MAPKs) [120]. In cardiac cells, these molecules have been linked to apoptosis, ischemia-reperfusion injury or ischemic heart failure [121], but it is unclear whether they are also involved in sepsis or processes such as calcium reuptake by the SR. 

#### Metalloproteinases 

The matrix metalloproteinases (MMPs) are a large family of zinc-dependent endopeptidases whose main property is to degrade extracellular matrix components. Its activity was found increased in a variety of cardiovascular diseases, including acute or chronic heart failure and atherosclerosis [122, 123]. The activation of MMP-2 appears to mediate the acute heart failure following ischemia-reperfusion by "cleavage" of Troponin I. Recently it has been documented its involvement in septic cardiomyopathy in animals and that its inhibition reversed it in LPS-induced septic shock [124]. In another animal study, the cardiac MMP-2 and MMP-9 were positively involved in cardiac heart rate and negatively correlated with Left Ventricular Stroke Work Index (LVSWI), and increased activity correlated positively with the occurrence of myocardial apoptosis [125]. 

#### NO, Peroxynitrite and Oxygen Free Radicals

Nitric oxide (NO) is produced by the NO synthases (NOS). These enzymes exist in three forms, the NOS-1 and NOS-3, called constitutive, and NOS-2, called inducible, with great vasodilator power but also to generate inflammation. In humans hearts with sepsis, increased expression of inducible NOS and significant amounts of peroxynitrite have been found, producing *in vitro* cardioinhibition at various moments of contraction and relaxation, either by production of NO as without it. In animal experiments, reversing some of the effects of LPS by administration of dexamethasone have succeeded, which appears to inhibit the induction of NOS 2, but not of NOS1 or NOS3, which suggests that NO cardiodepression is dependent of the induction and translation of a inducible protein [126, 127]. However, other studies found that myocyte depression and decreased intracellular calcium occurr early after the stimulation of IL-6, and TNF-α can block β-adrenergic effects without increasing the mRNA for NOS2, which in any case involve the NOS 1 and NOS 3 constituent. A recent theory advocates that NO acts as a second messenger, via nitrosylation of thiol groups of cysteine [128], and that the NOS-3 isoform found in the sarcolemmal membrane produces NO that modifies L-calcium channels, inhibiting calcium entry, and inducing relaxation of the myofibril. NO derived from NOS-1 in the SR nitrosyles ryanodine channel so that induces the calcium output from the SR resulting in the contraction. NO signals are rapid, reversible and affected by the oxide-reductive balance of the system [129, 130]. The NADPH oxidase and xanthine oxidase, coupled with oxidative phosphorylation, generate free radicals: superoxide, hydrogen peroxide and hydroxyl. At high levels, these reactive oxygen species are toxic through several mechanisms (direct damage to DNA, proteins and lipids, competition for thiols, etc.), that are irreversible and do unable to interact with NO, which breaks NO signaling system. Apparently, the NOS isoforms can interact directly with enzymes that cause production of reactive oxygen species (ROS), so that may occur uncontrolled amounts. Thus in animal models, it has been shown that animals deficient in a subunit of NADPH oxidase have less ventricular hypertrophy and interstitial fibrosis after exposure to angiotensin II [131], and other studies have found that inhibition of xanthine oxidase with allopurinol improved endothelial dysfunction parameters in smokers and diabetics. However, experiments in humans have not shown improvement with the xanthine oxidase inhibition in the onset of symptoms, contractility, or prognosis. In any case, inhibitors of the NO employeed (methylene blue, etc.) have proved ineffective [132]. 

#### Mitochondrial Dysfunction

Mitochondrial dysfunction seems to be linked to the severity and prognosis, both in patients and experimental animals [133]. In animals, reduced consumption of oxygen in the final stages of sepsis but not the initials and alterations in the activity of the electron transport chain of mitochondria have been reported. It seems that mitochondrial DNA is more susceptible to endotoxin-induced damage than nuclear DNA [134]. One theory even suggested that SIMD could represent a protective adaptation to reduce energy consumption during a state of low production of ATP by mitochondrial dysfunction, similar to the phenomenon of hibernating myocardium during ischemia [135]. 

#### Apoptosis and Cell Death

There is increasing evidence that apoptosis is involved in SIMD [136], probably by induction of substances involved in its start as caspases and mitochondrial cytochrome C, which may be in the mechanisms of myofilaments altered responses, contractile proteins fragmentation and sarcomere structure disorganization [137]. Some strategies to inhibit apoptosis in animal models of sepsis appeared to have improved contractile function [138]. The relatively quickly recovery of cardiac function in SIMD survivors make unlikely that this mechanism of apoptosis was critical. 

## DIAGNOSIS

In the 2001 International Sepsis Definitions Conference [139], hemodynamic variables diagnostic criteria of severe sepsis were defined: Sepsis-induced hypotension, as defined in the first ACCP / SCCM Consensus Conference as systolic blood pressure (SBP) <90 mm Hg, MAP <70, or an SBP decrease >40 mm Hg in adults or> 2 SD below normal for age [140], and introduced two new variables, mixed venous oxygen saturation (SvO_2_) >70% and cardiac index (CI) >3.5 L/min^-1^. The concept of hemodynamic failure of this Consensus Conference is still not entirely correct, since it does not differentiate between sepsis-induced circulatory dysfunction and sepsis-induced cardiac dysfunction. 

### Invasive Hemodynamic Monitoring Systems

Although studies based on transpulmonary thermodilution method provide key hemodynamic variables as extravascular lung water, extralung water index and intrathoracic blood volume, can detect incipient pulmonary edema and its use has been shown reduce the duration of mechanical ventilation and, therefore, admission in the ICU [141, 142], the most recent studies mainly used echocardiography. The FloTrac / Vigileo system, which estimates the aortic impedance by analyzing the blood pressure curve and requires no calibration, proved to be less reliable in hemodynamic instability, abrupt changes in arterial tone and hyperdynamic situations, but has not been evaluated directly in patients with severe sepsis, in which there are all these features that can decrease the accuracy of the measurement. In a recent study by Monnet and coworkers with 80 patients in a state of septic shock in which cardiac output was compared measured by PiCCO and Vigileo systems, the PiCCO system showed greater accuracy to detect changes in CO produced by volume expansion as those induced by norepinephrine, but we must emphasize that many calibrations were performed, which in daily practice aren´t done, and could subtract reliability of those results. The inaccuracy Vigileo system was higher with increasing change in systemic vascular resistance [143]. 

### Electrocardiograph

The SIMD can be accompanied by specific changes in the electrocardiogram (ECG), which can be very similar to those occurring during acute coronary syndromes [22], including ST segment depression or elevation, Q wave appearance, new left bundle branch block, peaked T wave, lenghtening of the QTc interval or positively J waves (Osborn wave) appearance [144]. The ECG may suffer temporary modifications, with an initial predominance of ST segment elevation followed by appearance of a Q wave. These ECG changes may disappear with time [145]. Interestingly, the ECG may suggest the diagnosis of myocardial dysfunction.

### Echocardiography

The same year that Parker described the SIMD, Ozier and coworkers showed in two-dimensional echocardiography [146]. Annan and coworkers in 2005 proposed the SIMD as a diagnostic criteria of severe sepsis, paying special attention to echocardiographic studies in septic patients, due to it is a rapid and non-invasive study that can be done at the bedside [1] (Table **3**). 

Echocardiography is a first-line technique for hemodynamic evaluation of patients with hemodynamic instability. In patients with severe sepsis or septic shock, early goal-directed optimization of CO by intensive fluid therapy has been shown to reduce morbidity and mortality [1, 140]; however it is also known that not all patients respond equally and that excessive fluid therapy can lead to respiratory failure. Traditional parameters estimatory of preload such as central venous pressure and pulmonary artery pressure, have not proven to be reliable in predicting fluid responsiveness; however, echocardiography is presented as a non-invasive alternative that can offer parameters such as respiratory variation of the inferior vena cava diameter and stroke volume (Fig. **4**). The analysis of the respiratory variation of the inferior vena cava diameters has demonstrated high and significant correlation with the increase in CO after the bolus [147, 148]. Schefold group, in a study with 30 severe sepsis and septic shock mechanically ventilated patients, found significative correlation between inferior vena cava diameters, central venous pressure, extra-lung water index, intravascular blood volume index, intrathoracic blood volume index, intrathoracic thermal volume and even the PaO_2_/FiO_2_ oxygenation index [149]. 

In severe sepsis or septic shock patients with positive-pressure mechanical ventilation, measurement of central venous pressure, pulmonary capillary wedge pressure or RV diastolic diameter can bias the analysis of the response to fluid therapy because intrathoracic pressure is increased and, therefore may decrease venous return and impair stroke volume [150]. However, it has been shown that dynamic measurement such as the respiratory variability of arterial pulse pressure [151], and transesophageal echocardiography studies of stroke volume variation [152] or respiratory variability diameter of the superior vena cava [153], can faithfully reproduce the response to fluid therapy in this group of patients. 

**Fig. (3):**
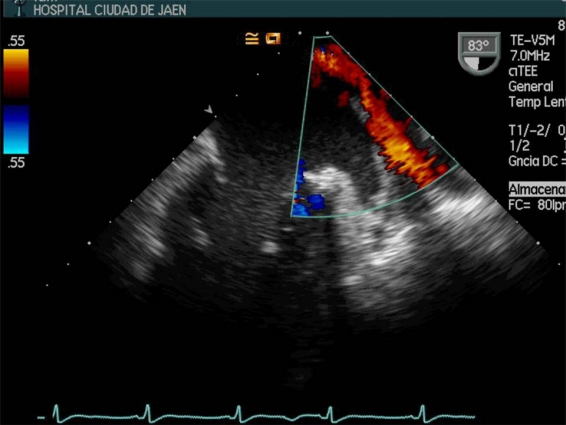
Transesophageal image projection which shows the pulmonary veins flow. Quantifying the systolic filling fraction, left atrial pressure can be calculated. (LA: Left atria. LV: Left ventricle. LAp: left atrial appendage).

**Fig. (4):**
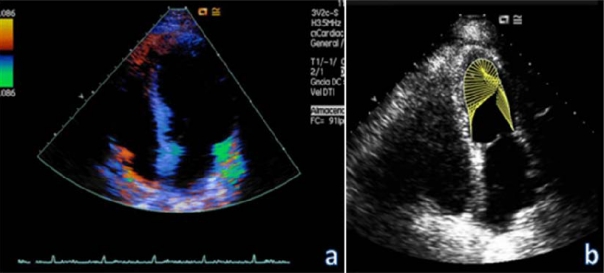
Left ventricular segmental contractility alterations. Evaluation by quantitative tecniques based on tissue Doppler image (**a**), or the velocity vector analysis (**b**).

**Fig. (5):**
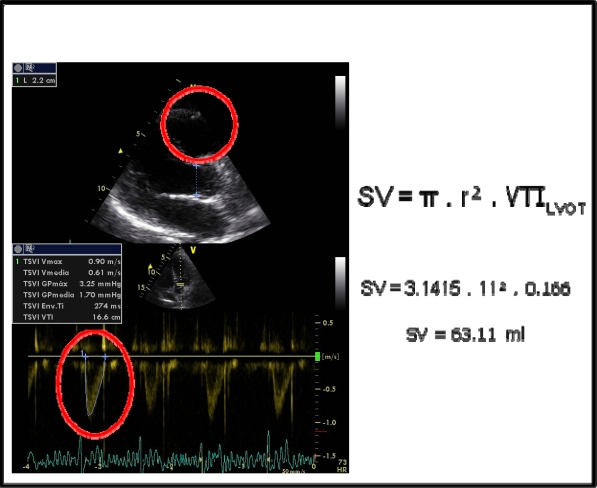
Stroke volume echocardiographic calculus: According to the equation SV= π x r² x VTI_LVOT_ = 59 ml (Normal). (SV= systolic volume, VTI_LVOT_= Left ventricular outflow tract velocity-time integral, r = left ventricular outflow tract ratio).

Regarding the analysis of ventricular contractility by echocardiography, it is known that some patients experience segmental contractility alterations, with hypokinesis mainly at the apex and basal LV segments [154, 155] (Fig. **5**). In a descriptive study with 33 myocardial dysfunction patients without previously known cardiac disease, we [145] found segmentary contractility disturbances in all the patients, with septoapical hypokinesia in 57% of the patients, akinesia in 28.6% and dyskinesia in 14.3% (Fig. **3**). The improvement in the LVEF over time was accompanied by a progressive and statistically significant improvement of segmental contractility alterations (p=0.0001). The analysis of global contractility by calculating the left ventricle ejection fraction (LVEF) has several limitations. The LVEF is clearly influenced not only by LV contractility, but also by the state of preload and afterload that are frequently altered in septic patients; however, LVEF measurement has proved to be very sensitive to changes in contractility when ventricular function is decreased. As in ischemic heart disease, the Teicholtz method is not the most appropriate when there are alterations of segmental contractility. 

Noninvasive assessment of diastolic filling by Doppler echocardiography provides important information about LV status. The ratio of mitral velocity to early diastolic velocity of the mitral annulus (E/E') by tissue Doppler imaging that combines the influence of transmitral driving pressure and myocardial relaxation are associated with invasive measures of diastolic LV performance, and has shown to predict the mean left ventricle diastolic pressure (M-LVDP). In shock patients preferences in the measurement site of Doppler tissue imaging E 'maximal velocity (at lateral or septal mitral annulus) has not been shown [156]. A ratio E/E '<8 accurately predicted normal M-LVDP, and E/E'> 15 identified very high M-LVDP. Wide variability was present in those with E/E' from 8 to 15 [157]. The ratio E/E' has proven to be predictive of left ventricular dysfunction in experimental models [158], failure of weaning from mechanical ventilation [159], and prognostic value in septic patients [160], and in critically ill patients in general [161]. It has also shown to provide better discrimination between hospital survivors and non-survivors than cardiac biomarkers (Brain Natriuretic Peptide, N-Terminal pro-Brain Natriuretic Peptide and cardiac troponin T) [162]. Existing evidence suggests that monitoring the ratio E/E 'could be one more parameter to guide the initial fluid resuscitation of septic patients and indicate the optimal moment to start the vasoactive treatment. It is striking how little literature on the use of tissue Doppler techniques for assessing myocardial dysfunction. Its future employment may give us more information. 

The right ventricle can also be studied by echocardiography with capacity of providing assessments of RV function similar to pulmonary artery catheter [163]. Size, wall thickness, contractility, RVEF and abnormal movements of the interventricular septum can be objectified. The RVEF is usually estimated qualitatively because the RV shape is not cylindrical unless there was availability of 3D echocardiography or radionuclide angiography. Recently, the Tricuspid Annular Plane Systolic Excursion, cardiac Magnetic Nuclear Resonance [164-166], and the Tei index [167-168], have gained value. The abnormal movements of the interventricular septum as septal flattening and paradoxical movements may suggest the existence of higher diastolic pressures. Myocardial perfusion echocardiography has been recently suggested the for differential diagnosis with acute coronary syndromes, which has revealed, in a patient with septic shock secondary to pneumonia with ECG changes, cTnI rise and disturbance of segmentary contractility, normal perfusion in areas of both normal and anormal contractility, suggesting that the cardiac presentation that was more likely to be a SIMD. Percutaneous coronary angiography showed no significant coronary artery disease [169]. Echocardiography is probably the most useful method for the diagnosis and management of patients with SIMD, however, requires that the modern intensivist was trained in the technique [170]. 

Dobutamine stress echocardiography has been used as a prognostic indicator in septic shock. Vallet's group [171] studied the oxygen supply and uptake responses to a 60-min dobutamine infusion (10 micrograms / min / kg) for 60 minutes in a group of 50 septic patients with normal blood lactate concentrations. Responders were called to the test who had a >15% increase in oxygen uptake from the time immediately before to 1 hour after. Mortality rate observed in the responders (8.7%) was significantly less than that rate in nonresponders (44.4%). Rhodes and coworkers [172] did a prospective and interventional clinical trial which studied the response to intravenous infusion of dobutamine at the same dose that the study by Vallet´s group and for an hour also, after the septic fluid replacement in 36 patients. Patients who were able to increase his or her oxygen consumption by >15% were designated a responder to the test. Interestingly, responders to this test had a hospital mortality of 14%, whereas nonresponders had a mortality of 91% (p <0.01). We evaluated the possibility that myocardial dysfunction was not fully reversible by conducting stress echocardiography in a group of 33 patients (28 septic patients) with mean age 41 years [173]. One month after hospital discharge, all the echocardiographic alterations had normalised. At six months, the dobutamine stress echocardiography showed the presence of akinesia or dyskinesia of the septoapical segments in eight patients. These disturbances of segmental contractility were detected at 10 mg/kg/min in six patients and at 15 mg/kg/min in the other two. Stress echocardiography was repeated in these eight patients at two years and the same disorders of segmental contractility persisted in all of them at a dose of 10 mg/kg/min. Based on the resolution of systolic function, RMD is thought to be totally reversible, but diastolic function has not been studied in such detail. Furthermore, we don´t know whether these patients are more susceptible to developing further episodes or their possible response to stress. Jellis and coworkers [174], in a recent study, suggested diffuse reactive fibrosis by cardiac magnetic resonance in non-ischemic pathologies which cursed with myocardial dysfunction. The possibility that myocardial dysfunction is not totally reversible in all patients should be investigated.

### Impedance Cardiography

The impedance cardiography (ICG) is a non-invasive method that converts changes in thoracic impedance in volume over time. It can make continuous measurements of SV, CO, systemic vascular resistance, left cardiac work, acceleration of contractility index and left ventricular ejection time among others. Napoli and coworkers [175], did an observational study with 56 patients with sepsis in the Emergency Room and used ICG during resuscitation guided by the usual static parameters (CVP, SvO_2_ and lactate). The acceleration of contractility index (ACI) measured by bioelectrical impedance was significantly lower in patients who died (71 l/s^2^ vs 123 l/s^2^), it was related to the left cardiac work index (r = 0 , 63, p = 0.01), and only predicted hospital mortality (AUC 0.70, p = 0.01). An ACI less than 40 l/s^2^ was associated with a hospital mortality rate 9 times higher than the control group. The ACI was not associated with lactate levels >4 mmol/l (p = 0.24), PVC (p = 0.56) or the fluid administered (p = 0.18). In this study we can conclude that the analysis of the ACI may be a measure of contractility independent of preload and that its reduction can be attributed to segmentary contractility alterations. Larger studies would be needed in which consider whether ACI could have predictive value to guide resuscitation of sepsis based on the patient's myocardial response. 

### Laboratory Test

The hormone B-type natriuretic peptide (BNP) is produced by ventricular myocytes. Main stimulus for BNP release is primarily increased ventricular wall stress [176], and several studies have shown that plasma BNP may be elevated in patients with septic shock [177, 178]. Both N-terminal proB-type Natriuretic Peptide (NT-proBNP) and BNP have been shown to possess strong predictive values for cardiovascular events [179,180]. In a prospective study of 49 patients with shock, mainly noncardiogenic, analysis for BNP was performed. In multivariable analysis, a BNP concentration in the log-quartile was associated with higher mortality (p <0.001) [181]. Another descriptive study of 78 critically ill patients found similar results in the subgroup of septic patients [182]. Post and coworkers [183] in a cohort of 93 patients with septic shock divided into two groups, one with normal LVEF at 3^rd^ -5^th ^day of ICU admission (n = 38) and another with LVEF <50% (n = 55), the authors analyzed the behaviour of plasma BNP with echocardiographic monitoring. They found that the lower was the LVEF higher was the plasma concentration of BNP (p <0.05), this difference was most evident after 5 days (86 pg / ml in patients without LV dysfunction vs. 699 pg / ml in patients without it, with p <0.0005). In addition, patients with LV dysfunction and elevated plasma levels of BNP were associated with increased morbidity and mortality, indicating that the plasma concentration of BNP could be used as a prognostic marker of septic shock, at least for the short term, given that the analysis of the curves ROC of the scores APACHE II and SOFA of these patients showed that the plasma concentration of BNP had significant predictive value for survival at 30th day. (APACHE II <24 points: NPV 61%, AUC 0.494, p= 0.88, sensitivity 18% and specificity 89%; SOFA <15 points: NPV 62.4%, AUC 0.493, p= 0.90, sensitivity 15.8% and specificity 96%). This study excluded patients with previously known cardiac disease who may have basal plasma BNP levels high. However, these patients are usually admitted to the ICU and in them, therefore, measurement of plasma BNP aren´t useful as a prognostic marker. Recently Hartemink and coworkers [184], in a prospective case-control study analyzed the plasma levels of NT-proBNP in 18 septic patients before and after colloid fluid loading. They found that NT-proBNP is an independent marker of greater systolic cardiac dysfunction, irrespective of filling status, and is a better predictor of fluid nonresponsiveness in septic vs. nonseptic, critically ill patients. 

Nowadays, it's possible to identify with more accuracy the septic patients whose myocardium has been compromise by assessing troponin (cTnI) levels, which proved to be a highly sensitive and specific marker of myocardial injury in sepsis. The mechanisms underlying cTnI release in patients with sepsis are still unknown. Several hypotheses have been developed that attempt to explain the increase in cTnI. One theory is that sepsis produces coronary hypoperfusion and ischemia; however, it has been shown that in patients with normal coronary arteries may also raise the cTnI [185]. Another theory could be that the sepsis would result in an increased platelet reactivity forming microthrombus in the circulation which also produces myocardial ischemia, but it has been recently refuted [186]. In some septic patients, elevated cTnI levels may indicate bacterial myocarditis [187]. Theories to contrast are the reversible myocardial membrane leakage and cytokine mediated apoptosis. Fernandes Jr and coworkers [11], in a single center study of 10 septic patients without previously known heart disease showed that all patients whose LVEF was <50% had elevated cTnI levels (kappa = 0.61, p = 0.035). Another similar study of Ver Elst´s group [188] showed that 78% of cTnI+ patients had reduced LVEF compared with 9% of cTnI- individuals. The elevation of cTnI could be related to the duration of the hypotensive episode in critically ill patients. In a prospective study of 19 patients with severe sepsis or septic shock and 12 patients with hypovolemic shock [187], markers of myocardial injury were studied (cTnI, CK, CKMB and myoglobin). In the group of patients with severe sepsis or septic shock cTnI increased in 11 of 19 patients while it rose in all patients with hypovolemic shock. The more time was hypotensive episode greater was the increase in cTnI (moderate hypotension: median 1.16, quartiles 0.55-3.44 ng / ml, severe hypotension: median 8.53, quartiles 1.1-20.7 ng / ml, p <0.05). Abnormal levels of cTnI were more frequent in non-survivors than in survivors (p <0.05). In a prospective study of 20 septic patients [189], levels of cTnI were analyzed, being high in 85% of patients and in these, coronary angiography, necropsy or stress echocardiography just found coronary artery disease in 41% of patients. A high percentage of patients with elevated cTnI (41%) had positive blood cultures for *Streptococcus pneumoniae*, but it has shown no causal link at this time. Mehta and coworkers [190], in a single center prospective study with 37 patients with severe sepsis, noted that 16 (43%) had cTnI+ and that these were associated with higher need for inotropic or vasopressor support (p = 0.018), higher APACHE II score (p = 0.004), higher incidence of segmentary Abnormalities of wall motion on echocardiography (p = 0.002) and lower LVEF (p = 0.04). The highest incidence of segmentary wall motion abnormalities in patients with cTnI + not found in other studies [191]. In several studies, cTnI elevation has been associated with worse prognosis [190, 191]. John and coworkers [191] hypothesized that drotrecogin alfa activated (DrotAA) treatment could improve outcomes in severe sepsis patients who have elevated troponin. They made a single center study with 105 patients with severe sepsis, and identified 48 (46%) with cTnI +. These were associated with higher mortality (52% in cTnI+ group and 30% in cTnI- group). DrotAA treatment significantly reduced mortality in patients with cTnI + (30% in cTnI+ patients treated with DrotAA and 72% in untreated cTnI+ patients). Recently the same author conducted a study using a subset of the PROWESS trial severe sepsis patients (n = 598) [193, 194]. This is the largest volume study sample (n = 598) that analyzes the prognostic value of cTnI in patients with severe sepsis. In 75% of the cases showed elevated cTnI, similar to other published studies that found in 43-85% of patients. The cTnI was shown to have independent prognostic value for mortality (p = 0.014, odds ratio 2.02, 95% confidence interval 1.15-3.54), being the 28-day mortality higher when the cTnI was positive (32% vs 14% p = 0.0001). However, cTnI was not a predictor of the survival benefit observed with DrotAA administration. 

Yucel and co-workers [195] did a prospective study of 40 patients with severe sepsis and mechanical ventilation, and assessed serum levels of ANP, BNP, cTnI and C-reactive protein (CRP) at ICU admission, and two days later of ICU discharge. Except for cTnI and CRP on day 1, the four parameters were significantly powerful to discriminate non-survivors on all days (AUC 0.731 to 1). BNP was the most powerful diagnostic parameter on all days (AUC 1).

## TREATMENT

Current standard treatment for sepsis include infection control and optimization of hemodynamic parameters (SvO_2_, mean arterial pressure (MAP), diuresis, CVP and hematocrit) by fluid replacement, vasoactive, transfusional therapy, inotropes, steroids or human recombinant protein C. These recommendations received a grade B recommendation (based on a single level I study), and are based on the study of Rivers [140], which was a single center study with small sample size in which the interventions were not performed blindly, which introduced the possibility of bias, and not touched a single therapeutic measure, but a protocol that included various diagnostic and therapeutic measures, which did not show that each of these measures were effective. Under these conditions, recommending adoption of all of them collectively is risky. In fact, many of the individual measures used are of questionable utility and effectiveness [151]. 

These treatments, in 10-20% of patients, fail to normalize the hemodynamics of patients with septic shock, existing high probability that the cardiac output was diminished by SIMD. There is no known way to mitigate the SIMD, although it begins to consider the need to study the administration of a cardioprotective therapy to critically ill patients, which could have a special role for beta-blockers, inhibitors of the enzyme converting (ACE) inhibitors, calcium channel blockers and statins [196]. Nor is there consensus for the treatment of diastolic dysfunction in the SIMD, although the evidence in similar circumstances such as heart failure, also suggests that ACE inhibitors, antagonists of angiotensin-II receptor, and beta blockers have a potential benefit [197]. 

In this section we will discuss various therapeutic strategies proposed in the treatment of SIMD, many recently published and in experimental period. We state that the treatment of SIMD remains a challenge almost 30 years after its characterization. 

### Vasoactive and Inotropic Therapy

There has been longstanding debate about whether one catecholamine vasopressor agent is superior to another. Depending on the effect of each catecholamine on its receptor (α-adrenergic vasoconstriction promoting receptors, β1-adrenergic receptors increasing heart rate and myocardial contractility, and β2-adrenergic receptors causing peripheral vasodilation) a treatment strategy that takes into account both blood pressure and tissue perfusion should be chosen [198]. 

Since the introduction of the protocols of the Surviving Sepsis Campaign, several observational and controlled trials have been published that reconsider the "*one-size-fits-all*" approach to a multimodal approach in vasopressor selection [199]. *Norepinephrine* or *dopamine* are the first choice of vasoactive drugs for hemodynamic support of septic shock after correcting any hypovolemia [200]. Norepinephrine may be more effective than dopamine in reversing hypotension in patients with early septic shock [201, 202]. In a recent study Hamzaoui and coworkers [203], with 105 patients in septic shock, found that early use of norepinephrine in situations of severe hypotension was able to increase cardiac output by increasing preload and the cardiac contractility, and when MAP was achieved ≥ 75 mm Hg, it failed to produce this effect. The authors did not examine the effects of norepinephrine in the long term. In a single center study with 26 patients on dobutamine-resistant septic shock [201], classified into two groups, treated with dobutamine were associated with norepinephrine or norepinephrine alone, showed that the addition of norepinephrine to dobutamine significantly improved cardiovascular function in patients with septic shock who were adequately volume-resuscitated but resistant to dobutamine used alone (increased CI and SVI and increased SVRI by 40%), suggesting that this combination therapy could be especially attractive in the setting of sepsis. The hemodynamic study was performed with pulmonary artery catheter and thermodilution method, without echocardiography. This positive effect of norepinephrine should be taken with caution because in a study by Vieillard-Baron and coworkers [9], which used echocardiography, 34% of their septic shock patients, previously nonhypokinetic, global LV hypokinesia occurred after 24-48 hours of continuous norepinephrine infusion. The proposed measures to reduce excessive adrenergic stress in patients with severe sepsis and septic shock are the control of temperature and heart rate, adequacy of sedation and analgesia, use of levosimendan and infusions of hydrocortisone and / or arginine-vasopressin [203]. Accumulating evidence suggests that norepinephrine may be effective in the initial stages SIMD but long term may be deleterious by sympathetic overstimulation. 

*Dopamine* may be useful in patients with SIMD because increases MAP in patients who remain hypotensive after optimal fluid therapy by increasing cardiac index. Its disadvantages are that it is very tachycardic and can be very arrhythmogenic [204, 205], although was not associated with increased mortality in patients with shock [206]. Recently De Backer and coworkers [207] in a multicenter and randomized trial involving 1679 patients in a state of shock, compared the results of treatment with dopamine or norepinephrine and found that patients who were treated with dopamine had higher 28 days- mortality (52.8%) than those treated with norepinephrine (48.5%), but it did not reach statistical significance (p = 0.10). In the analysis of a subgroup of patients in the study they noted that treatment of cardiogenic shock with dopamine was associated with higher mortality (p = 0.03). 

For the above reasons, in patients with persistently low cardiac output despite adequate LV filling pressure (or clinical assessment of adequate fluid resuscitation), *dobutamine* is the preferred treatment recommended by current clinical guidelines [208], which has proved enhancing cardiac index (from 12% to 61%), ventricular stroke work index (from 23% to 58%), right ventricular stroke work and oxygen delivery index (DO_2_I), at the expense of an often significant increase in heart rate (from 9% to 23 %) [209-211]. 

### Vasopressin and Terlipressin

Vasopressin (AVP) and terlipressin are increasingly used as adjunct vasopressors in the treatment of catecholamine-resistant septic shock [212]. The deficit of vasopressin and downregulation of vasopressin receptors in severe sepsis and septic shock [213], would justify their use. Russell and coworkers [214], found that vasopressin levels were extremely low in septic shock (median 3.2 pmol / L), and increased during low dose vasopressin infusion (0.03 U / min) to about 74 pmol / L (6 hours) and 98 pmol / L (24 hours). 

The V2 agonistic effects of AVP may exert favourable effects on the hepatosplanchnic, renal, pulmonary and coronary circulation. Terlipressin has more activity on V1 receptors and may increase the blood pressure more potently avoiding rebound hypotension. The use of each drug is usually according to local availability [215]. In patients with catecholamine-dependent septic shock, terlipressin increases MAP doing possible to reduce norepinephrine requirements but decreasing cardiac output, oxygen index and SvO_2_, by baroreceptor activation, effects that can be counteracted by dobutamine. In the study by Morelli and coworkers (DOBUPRESS study) [216], the authors compared the terlipressin-dobutamine combination treatment in a group of 20 patients with catecholamine-dependent septic shock, and compared with other two groups treated with increasing doses of norepinephrine (control group, n = 20) and with terlipressin-norepinephrine (n = 19). There were no significant differences between the three groups in heart rate, MAP and LVSWI were higher in the group treated with terlipressin-dobutamine (p ≤ 0.001 and p = 0.006 respectively). While the control group needed progressively increasing doses of norepinephrine, in the other two groups the dose was reduced (both p <0.001), and even could be discontinued after terlipressin administration at the expense of high doses of dobutamine. In 35% of cases the dose of dobutamine was> 28μg/kg/min at 4 h to maintain SvO_2_ at baseline. In this group of patients adverse effects is expected to happen such as increased myocardial oxygen demand, tachyarrhythmias or decrease in MAP in the presence of hypovolemia. 

### Levosimendan

Considering that the SIMD is determined largely by a decrease in myofilament response to calcium, sensitization with levosimendan becomes an attractive therapeutic option. Levosimendan is a positive inotropic and vasodilator drug with demonstrated beneficial effects in acute heart failure and acute coronary syndromes [217-219]. Levosimendan has beneficial effects on both ventricles, independent of the beta-adrenergic signalling or changes in intracellular calcium concentration, by increasing contractile myofilament sensitivity to calcium. 

Levosimendan is a very experienced agent in animal models of induced sepsis [220-224], and we have very few human studies. The first publication that referred to the use of levosimendan in septic shock was a case report of Matejovic and coworkers in 2005 with a successful outcome [225]. Subsequently, Noto and coworkers [226] described the use of levosimendan in 2 patients with septic shock refractory to conventional treatment, and Powell and De Keulenaer [227] did the same with 6 patients. In all these studies, the authors found an improvement of hemodynamic status of patients, managing to reduce the need for treatment with catecholamines. There have been only two published clinical trials with levosimendan in sepsis. Morelli and coworkers [228], in a prospective case-control study with 28 patients with septic shock and persistent left ventricular dysfunction (LVEF <45%) after 48 hours of "conventional“ treatment including dobutamine up to 5 microg/kg per minute. 15 patients were treated with levosimendan (0.2 microg/kg per minute) and 13 remained with dobutamine (5 microg/kg per minute). An increase in SVI, CI and LVEF compared to baseline was observed. Treatment with levosimendan, without initial bolus, was associated with improvements in end-diastolic and end-systolic volume index (EDVI and ESVI), and LVSWI. In the second clinical trial reported, also led by Morelli [229], the authors studied the behaviour of levosimendan in a group of patients with septic shock and adult respiratory distress syndrome. 18 patients were treated with levosimendan and 17 with placebo. When the MAP was between 70 and 80 mm Hg, sustained with norepinephrine, the group of patients treated with levosimendan had increased CI, PVRI, RVESV and RVEF (all p <0.05). Large prospective, randomized clinical trials are now warranted. 

### Cardioprotective Therapy

Over 50 years of experimentation with *beta-blockers* in sepsis, since Berk studies in the 60s and 70s, have been insufficient for its inclusion in universal guidelines of sepsis treatment [230,231]. The benefits of beta blockade have been demonstrated in clinical trials of pediatric severe burn [232], patients with heart failure [233], severe trauma [234,235], traumatic brain injury [236], or perioperatively [237], demonstrating that beta-blockers are effective preventing ischemia, decreasing oxygen demand (reducing cardiac output up to 20% without worsening of oxygen utilization or increase lactate levels), and decreasing TNFα production [238], allowing for greater preserving cardiac function. As the evidence suggests that beta adrenergic stress is a major factor in the pathogenesis of SIMD [239], the use of beta-blocking agents could be benefitial. However, there is controversy as it may seem potentially deleterious administer a negative inotropic drug in a patient with SIMD. There are several experimental studies using beta-blockers in sepsis, having shown mortality reduction if commenced before a septic insult [240]. Landiolol treatment, an ultrashort-acting beta-blocker, was associated with significant reduction in serum levels of the inflammation mediator HMGB-1 and histological lung damage [241]. In humans, there is only scarce information regarding this issue. Gore and Wolfe [242] observed a 20% decrease in heart rate in septic patients with continuous esmolol infusion. Interestingly, no significant differences in hepatic blood flow or extremities were found. The authors hypothesized that esmolol could reduce the risk of myocardial ischemia without systemic consequences of hypoperfusion. Schmittinger and coworkers [243] did a retrospective study of 40 patients with septic shock of their institution who were treated with combined milrinone and enteral metoprolol. They found that treatment with beta-blockers was a way to “economize” cardiac function, resulting in a maintained cardiac index with a lower heart rate and a higher stroke volume index. In a retrospective study of 83 septic patients, of whom only were previously treated with beta-blockers 23, the authors didn´t show a significant association between beta blockers and increased mortality in septic patients [244], but the overall mortality was 10% higher in the group exposed to beta-blockers, although this difference was not statistically significant, it showed a trend toward worse outcome. 

Both *simvastatin* and *fluvastatin* have shown clear antimicrobial [245], antiviral and antifungal effects. The largest study on the usefulness of statins administered in septic patients was performed on a cohort of patients over 65 years admitted for acute coronary syndrome, acute stroke or who required revascularization and survived three months after hospital discharge. In this cohortwere studied 69 168 patients (34 584 patients in each group). The incidence of sepsis was lower in patients receiving statins (71.2% versus 88% events per 10,000 person-year, risk reduction 19% (RR 0.81, 95% CI 0.72 to 0.91). The protective effect against sepsis persisted in high-risk subgroups such as diabetes, chronic renal insufficiency or a history of infection. Also there was a significant reduction in severe sepsis (RR 0.83, 95% CI 0.70 - 0.97) and in the "fatal sepsis” (RR 0.75, 95% CI 0.61 to 0.93). However, they could not find any benefit with the administration of other non-statin lipid-lowering agents [246]. Other observational studies detected similar results [247,248]. Through an evaluation of 22 studies, including 177 260 patients, the effect of statins on sepsis were studied. 19 studies cohorts were evaluated (seven prospective and 12 retrospective), 2 case-control studies, plus a randomized controlled trial. The effectiveness of the administration of statins was studied in nine studies involving patients with either sepsis in 4 studies included patients with community-acquired pneumonia in three studies of patients with bacteremia, and in three postoperative patients were included. Mortality was lower among statin users (3 of 6 studies of sepsis, 5 of the 6 studies of community-acquired pneumonia and 2 of the 3 studies of bacteremia). In four studies there were no differences in mortality and in one study there was an increase in mortality over patients who received statins. In 5 of the 9 studies whose primary objective was to examine the risk of sepsis or infection in relation to the administration of statins, there was a reduction in risk among statin users, while the other studies found no differences. The majority of patients who were treated with statins in different studies, occurred more frequently more cardiovascular disease, greater comorbidity, more diabetes, hypertension, hyperlipidemia, renal failure and even higher APACHE II score. However, the authors found globally, and independently of the design of different studies (making a proper match, randomization, etc), that most studies suggested that statins had a beneficial effect on the outcome of infection, showing lower mortality, less hospital stay and fewer complications (especially those studies that propensity analysis were performed). However, most not-observational studies could not obtain firm conclusions [249]. Using a randomized double-blind study it was evaluated the incidence of severe sepsis and the inflammatory response to the administration of statins. A total of 83 patients with documented bacterial infection towards the administration of simvastatin or to placebo were randomized. Due to low recruitment, the study was stopped early, reaching no differences in the incidence of severe sepsis, even with administration of simvastatin showed a decrease in the levels of TNFα and IL-6 [250]. In addition to the potential benefit of statins in sepsis, it would be interesting to antimicrobial and anti-inflammatory effects of statins, given even early in the SIMD. 

Several experimental animal studies in rats have shown that the *dihydropyridines* such as manidipine, nicardipine and especially amlodipine, apart from its effect on calcium channels, could reduce levels of proinflammatory cytokines and the expression of inflammation-relevant genes TNFα and iNOS [251,252]. Xiao-Qiang and coworkers [252] showed that pretreatment with amlodipine attenuated the myocardial inflammation induced by LPS, prevented hypotension and decreased inflammatory cell infiltration in the myocardium, suggesting an effect of cardiac protection. It would be interesting to study the effects of dihydropyridines in an experimental model of sepsis already established. 

### Other Treatments at an Experimental Stage

*Intra-aortic balloon counterpulsation* (IABP) is expected to be succeeded in reducing dosage of vasopressor drugs and to increase the survival time of patients with SIMD, allowing the treatment started took effect. Therefore it would be a maintenance therapy of patients with SIMD. The insertion of the IABP should be early, since it needs 3-24 hours to achieve its full operation [253]. Pribble and coworkers [254], in an experimental animal model with newborn lambs 

infected with group B *Streptococcus*, showed that IABP could increase cardiac output and decrease pulmonary resistance. Despite the findings of Engoren and coworkers [255] in a porcine model of endotoxemic shock, in which IABP use did not have benefits, the group of Solomon [256], more recently, in an experimental canine model, found that animals receiving the highest bacterial dose, IABP improved survival time (23.4 +/- 10 hrs longer, p=0.003), and lower norepinephrine requirements (0.43 +/- 0.17 microg/kg/min, p=0.002) and systemic vascular resistance index (1.44 +/- 0.57 dynes/s/cm^5^/kg, p=0.0001) compared with controls. Although the complications associated with use of IABP are rare, the incidence may increase in the subgroup of patients with septic shock with renal dysfunction or disseminated intravascular coagulation. The "ideal patient" to use the IABC would be the septic patient with a highly depressed myocardial function and a not severely reduced SVR, as a bridging intervention to give causal treatment time to work [257]. To evaluate the use of ventricular assist devices in selected patients with SIMD with devices like the *Impella* should not be considered a "folly" [258]. 

*Apelin* was recently identified as the putative endogenous ligand receptor coupled to G protein, which is related to the angiotensin AT1 receptor, also called receptor like-1 angiotensin II (AGTRL1) by reverse pharmacology. It has been considered the most potent positive inotropic peptide, suggesting that a decrease in the concentration of endogenous Apelin plays an important role in the development of heart failure [259]. Pan's group recently demonstrated in an experimental model in rats, which were induced the development of severe sepsis and septic shock, that plasma and myocardial concentrations of Apelin and AGTRL1 decreased and that exogenous administration improved the cardiac dysfunction originated, with greater hemodynamic stability and less release of cytokines such as MCP-1 and IL-8 [260]. 

The infusion of *mesenchymal stem cells* (MSCs) is a potentially novel treatment for SIMD. Weil *et al* [261], in a murine experimental model, showed that MSCs could mitigate the SIMD modulating the systemic inflammatory response by decreasing levels of TNFα, IL-1β and IL-6 produced by host macrophages. There was also an increase in serum levels of IL-10, endogenous inhibitor of cytokine production. Administration of endotoxin produced a 3-fold reduction in cardiac contractility and in mice treated with MSCs contractility improved 1.5-2-fold, without finding any response in the control group. Unfortunately, cardiac function was not completely restored and the experiment was done with injection of endotoxin istead of polymicrobial inoculum, which would have been more applicable to the human model. 

Recently, Netthling and coworkers [262] studied the cardiac effects of *diazepam* (Type IV phosphodiesterase inhibitor) in a murine experimental model after-LPS-induced endotoxic shock. The authors found that different functional myocardial parameters (LVDP, + dP / dt, RPP) and coronary flow were significantly improved after administration (p <0.01). 

## CONCLUSION

Sepsis-induced myocardial dysfunction is being studied extensively. Probably, It could have greater clinical entity that is known. However, its pathogenesis as well as multiple is still unknown. Probably the best method of diagnosis is echocardiography, but this technique requires an adequate training of the modern intensivists. There is no adequate treatment, althoug it seems that might be necessary to seek a cardioprotective therapy and supportive measures, similar to those specified in cardiogenic shock, while the casual treatment of sepsis takes effect. 

## Figures and Tables

**Table 1.** Sepsis Definitions

**Systemic inflammatory response síndrome**	≥ 2 of the following:	Body temperature >38,5°C or < 35°C. Heart rate >90 beats per minute. Respiratory rate >20 breaths per minute or arterial CO_²_ tension <32 mm or need for mechanical ventilation. White blood cell count >12000/mm³ or <4000/mm³ or immature forms >10%.
**Sepsis**	Systemic inflammatory response syndrome + documented infection	
**Severe sepsis**	Sepsis and at least one sign of organ hypoperfusion or organ dysfunction:	Areas of mottled skin. Capillary refilling time ≥ 3 s. Urinary output <0,5 ml/kg for at least 1 h or renal replacement therapy. Lactate >2 mmol/L. Abrupt change in mental status or abnormal electroencephalogram. Platelet counts <100000/mL or disseminated intravascular coagulation. Acute lung injury or acute respiratory distress syndrome. SIMD (echocardiography).
**Septic shock**	Severe sepsis and one of the following:	Systemic mean blood pressure <60 mmHg (<80 mmHg if previous hypertension) after 20-30 mL/kg starch or 40-60 mL/kg serum saline, or pulmonary capillary wedge pressure between 12 and 20 mmHg. Need for dopamine >5 µg/kg per min or norepinephrine or epinephrine <0,25 µg/kg per min to maintain mean blood pressure above 60 mmHg (80 mmHg if previous hypertension).
**Refractory septic shock**	Need for dopamine >15 µg/kg per min or norepinephrine >0,25 µg/kg per min to maintain mean blood pressure above 60 mmHg (80 mmHg if previous hypertension).	

**Table 2.** Noncardiac Critical Illness where Reversible Myocardial Dysfunction can Appear

Neurogenic myocardial dysfuncion:
Subarachnoid hemorrhage.
Ischemic stroke.
Subdural hemorrhage.
Head trauma.
After electroconvulsive therapy.
Reversible posterior leukoencephalopathy syndrome.
Acute respiratory failure:
Upper airway obstruction.
Asthma.
Pulmonary embolism.
Acute lung injury,
Acute respiratory distress syndrome.
Anaphylaxis.
Trauma:
Pulmonary contusion.
Politrauma.
Hemorrhagic shock.
“Blast injury”.
Severe burn.
Strangulation.
Post-transplantation.
Sepsis.
Systemic Inflammatory Response Syndrome.
Pancreatitis.
Cardiac arrest.
Poisoning.
Rhabdomyolysis.
Hypertensive crisis / pheochromocitoma.
Thyroid disease.
Arrhythmias.
Hyperthermia / hypothermia.
Obstructive jaundice.
Emotional stress.
Malnutrition.
Hypocalcemia

**Table 3.** Studies of Right Ventricular Function in Sepsis and its Relationship with Survival

Authors	Year	n	Survivors Initial RVEF	Nonsurvivors Initial RVEF	Survivors Follow-up RVEF	Nonsurvivors Follow-up RVEF
Hoffman *et al.*	1983	9	0.29 ± 0.06	0.36 ± 0.15	0.35 ± 0.1	0.34 ± 0.07
Vincent *et al.*	1988	56	0.28 ± 0.09	0.21 ± 0.07	-	-
Dhainaut *et al.*	1988	23	0.32 ± 0.13	0.29 ± 0.11	0.31 ± 0.12	0.22 ± 0.11
Parker *et al.*	1990	39	0.35	0.41	0.51	0.39
Vincent *et al.*	1992	68	0.43 ± 0.16	0.31 ± 0.13	-	-
Vieillard-Baron *et al.*	2008	67	0.35 ± 0.19	0.31 ± 0.14	-	-
